# Evaluation of the Protoscolicidal Effects of Albendazole and Albendazole Loaded Solid Lipid Nanoparticles

**Published:** 2019

**Authors:** Shohreh AMINPOUR, Abdollah RAFIEI, Ali JELOWDAR, Maryam KOUCHAK

**Affiliations:** 1. Infectious and Tropical Diseases Research Center, Health Reaserch Institute, Ahvaz Jundishapur University of Medical Sciences, Ahvaz, Iran; 2. Department of Parasitology, School of Medicine, Ahvaz Jundishapur University of Medical Sciences, Ahvaz, Iran; 3. Department of Pharmaceutics, School of Pharmacy, Ahvaz Jundishapur University of Medical Sciences, Ahvaz, Iran

**Keywords:** Albendazole, Nanoparticles, *Echinococcus granulosus*, Hydatid cyst

## Abstract

**Background:**

Protoscolex plays an important role in the development of hydatid cyst. Albendazole is one of the most effectual protoscolicidal agents for averting the reappearance of this disease, nonetheless, its low solubility and its low intestinal absorption necessitates the presence of a drug carrier to enhance its efficacy. In this study, the effect of albendazole and its nano-form on protoscolices in cultured media and in vivo was evaluated.

**Methods:**

Microemulsion method was used to prepare the Solid lipid nanoparticles (SLNs) containing albendazole. Infected livers were collected from the Slaughterhouse of Ahvaz, Khuzestan in 2017. The protoscolices were stored in RPMI 1640 for one week, and their survival under the influence of albendazole and nano-albendazole on days 3 and 7 at concentrations of 250 and 500μg / ml was investigated. The live protoscolices exposed on day 3 at a concentration of 250 μg/ml were injected to mice for evaluation of pathogenicity. Three months later, after autopsy of the mice, the pathogenicity was evaluated.

**Results:**

Protoscolicidal efficacy was highest in both concentrations on day 7 for albendazole and on day 5 for nano-albendazole. Following the autopsy of the mice, cyst growth was reported in all mice, and only two mice from the albandazole loaded SLNs group did not have any cyst.

**Conclusion:**

Albendazole loaded SLNs showed a higher protoscolicidal property than the free form of this drug; therefore, the use of nano-formulation of this drug is recommended to prevent the onset of this disease.

## Introduction

Hydatidosis is one of the most common worm diseases worldwide (1) and is more widespread in temperate, mountainous areas where livestock is prevalent (2). Due to its geographical and climatic conditions, Iran is considered as a target of this disease.

The condition is complicated when cysts are torn, and a large volume of fluid is released which contains an enormous number of protoscolices lead to an anaphylactic shock (3). Since protoscolices are the main cause of secondary cysts (4), these cysts are formed thereafter. Treatments for hydatid cyst include surgery, PAIR (Puncture, Aspiration, Injection, and Respiration), and drug therapy. For calcified cysts, the patient is also monitored to follow the course of the disease. Drug therapy is used in cases where patients have several cysts in two or more organs and they are less than 5 cm in size. This method is also used to prevent the post-surgical recurrence of the disease (5, 6).

Many drugs have been tested in this regard, but most of them had a limited effect on disease control, and it was with the introduction of benzimidazoles in the 1970s when drug therapy became possible for this disease. The first widely used drug was mebendazole (7), but albendazole showed a better anti-worm effect (8, 9), such that the level of metabolites created from albendazole in the serum is up to 10 times higher than that of metabolites produced from mebendazole (10).

However, albendazole has its own disadvantages including low solubility in water, poor absorption, rapid metabolism as a protein, and its distribution to other non-target tissues (11). Consequently, several efforts have been made to resolve these problems. Ultimately, a strategy to overcome these problems was to use the system of carriers, including liposomes, cyclodextrins, and nanoparticles (12–14).

Among these carrier systems, SLNs have been used as an appropriate carrier system since the early 1990s (15). Compared to other colloidal carriers, these nanoparticles have advantages such as the ability to control drug release and drug targeting, increasing drug chemical stability, acting as a carrier for lipophilic and hydrophilic drug, and having mass production capability and completely sterilized (11).

Therefore, the use of protoscolicidal agents such as albendazole after aspirating cysts could be very effective in preventing secondary cysts (16–23). In this study, the effects of albendazole and albendazole loaded SLNs were investigated on protoscolices in vitro and in vivo.

## Materials and Methods

Compritol 888 ATO was bestowed by Gattefossé Co. (France), albendazole was purchased from Damloran Razak Co. (Iran).

### Preparation of SLNs containing albendazole

Microemulsion method was used to load the drug on SLNs. First, 5 gr of Compritol 888 ATO was melted at 75 °C. Then, 0.5 gr of albendazole was added to it and mixed completely. At the same time, in another beaker, 100 mg of deionized water containing 1% tween 80 and 1% polyvinyl alcohol (PVA) was heated to 80 °C and slowly added to the previous mixture. The contents of the second beaker were slowly added to the first beaker sonicated after 4 min using a sonication bath (Elma Hans Schmidbauer GmbH & Co. KG-Germany). It was then homologated using a homogenizer (IKAR-Werke GmbH & Co. KG-Germany) at 12,000 rpm for 10–15 min. Simultaneously with the homogenization of this mixture, 100 ml of deionized water at 4 °C was slowly added to obtain the suspension containing nano-particles. The suspension was centrifuged for 5 min at 4000 gr. Subsequently, the supernatant was passed through a 2 μm filter using a pump. The obtained suspension was centrifuged at 30000 gr in two turns, 30 min each. The SLNs were isolated, and the supernatant was used to determine the amount of unloaded drug with a UV spectrophotometer at 296 nm(24).

All of the above steps were also performed to prepare the blanked nanoparticles used as controls, with the difference that albendazole was not added to the oily phase. Ultra Violet ray was utilized to sterilize the product.

### Characterization of albendazole loaded SLNs

#### Particle size, polydispresity index and zeta potential

The Photon Correlation Spectroscopy (PCS ) method was employed for measuring the mean particle size and polydispresity index (PDI) by a ScatterScope (Qudix-South Korea), and the zeta potential parameter was also determined by the Nano ZetaSizer (Zen 3600, Malvern Instrument Ltd, Malvern, UK).

### Atomic force microscopy (AFM)

The morphological characteristics of the nanoparticles were determined at room temperature using an atomic force microscope (Nanowizard II; JPK Instruments; Germany AFM).

### Drug Entrapment Evaluation

To determine the loading efficiency of albendazole on SLNs, a precipitated top-level solution which contained free drug and water was used (25).

### Collecting and preparing hydatid cysts

Infected livers were collected from the Slaughterhouse of Ahvaz, Khuzestan in 2017, and transferred to the lab of the Department of Parasitology, Faculty of Medicine, Ahvaz Jundishapur University of Medical Sciences. First, the external surface of the liver was washed with water and then washed with the normal saline and was immediately transferred to be placed under the sterile conditions. To collect protoscolices, the cysts surface were applied with 70% alcohol, and then hydatid cyst fluid was transferred to 50 cc sterilized falcon tubes. These tubes were fixed to settle the protoscolices. Thereafter, they were washed three times with phosphate buffered saline (PBS) and then washed twice with the main RPMI 1640 medium, each time having a fifteen minute rest period for sedimentation of protoscolices for the specimens (26).

In the next step, to detect the protoscolex viability, flame cell activity and protoscolex movements were investigated using a microscope. Eosin staining was also used as a method for determining the viability of protoscolices. Since the distribution of protoscolices in culture media is essential for drawing accurate conclusions, the number of protoscolices per unit volume was calculated. At least 2,000 live protoscolices are needed for intra peritoneal injection to any mice.

### Grouping of the cultures and the mice

The culture media were divided into four groups. The first group included the control group. Since methanol and dimethyl sulfoxide (DMSO) (the final concentration of DMSO should not be more than 0.1%) were considered as a solvent for albendazole due to its very low solubility, they were used as controls in the albendazole group, while for the nanoalbendazole group, drug-free SLNs were added as controls to the culture medium containing protoscolex.

The second group was intended to receive the drug. The first subgroup received albendazole and the second subgroup received nanoalbendazole. The concentrations of 250 μg/ml and 500 μg/ml of the drug were used in the culture medium. In all four groups, RPMI 1640 medium, 25% embryonic serum, 42% D-glucose and 45% yeast extract were used. Moreover, to prevent microbial and fungal contamination of the culture medium, 200 μg/ml of Pen-Strep and 1μg/ml of amphotericin B antibiotics were used.

The percentage of protoscolex survival was evaluated on days 1, 2, 3, 5 and 7. On the third-day experiment group, 0.5 cc of the protoscolices at 250 μg/ml concentration, containing at least 2,000 live protoscolices was injected intraperitoneally to BALB/C mice aged 6–8 wk and weighing 20–25 gr to evaluate the pathogenicity of the protoscolices exposed to the drug. The mice were kept in suitable conditions in terms of temperature, humidity, light, and nutrition for three months.

After three months, in order to investigate the pathogenicity of the drug-exposed protoscolices, the mice were anesthetized and underwent autopsy, and their abdominal cavity and other organs of the body were evaluated for the formation of cysts.

### Ethics

All procedures were done in accordance with the regulations for keeping animals in the laboratory, approved by the Ethics Committee of Ahvaz Jundishapur University of Medical Sciences to avoid animal poisoning and unnecessary harms. Isolated cysts were examined for weight and size using a digital scale and a caliper, respectively.

### Statistical analysis

The data were analyzed using SPSS ver. 19 (Chicago, IL, USA). The one-way ANOVA and descriptive statistics such as frequency calculation were used for data analysis.

## Results

### Average particle size and zeta potential

The particle size of albendazole-loaded SLNs and blanked SLNs was less than 40 nm. The PDI in both samples was also calculated to be 0.97. Zeta potential for albendazole loaded SLNs and for blanked SLNs was −20.8 and −21.5, respectively

### Drug loading and drug content of nano-particles

The loading efficiency was 94%, and the loading capacity of the nanoparticles was 6.2%.

### Morphological characteristics of nanoparticles

**Fig. 1:**
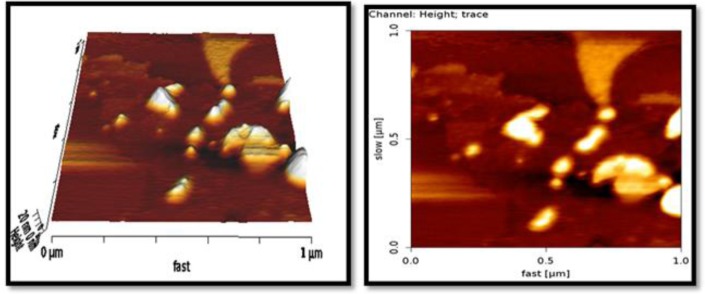
AFM Microscope images from ABZ-SLN

The results of albendazole loaded SLNs by AFM showed that the particles had spherical or semi-spherical forms and were less than 20 nm in size (Fig. 1).

### The protoscolicidal effect of the drug under laboratory conditions with different concentrations and time periods

**Fig. 2:**
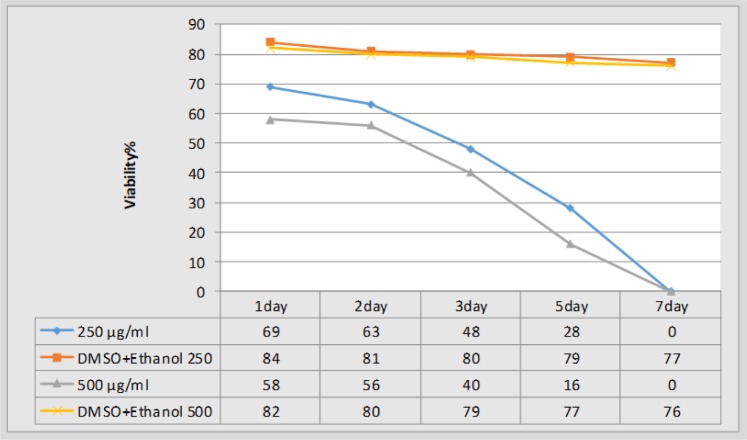
The Survival of protoscolex at different time periods and various concentrations of albendazole

**Fig. 3:**
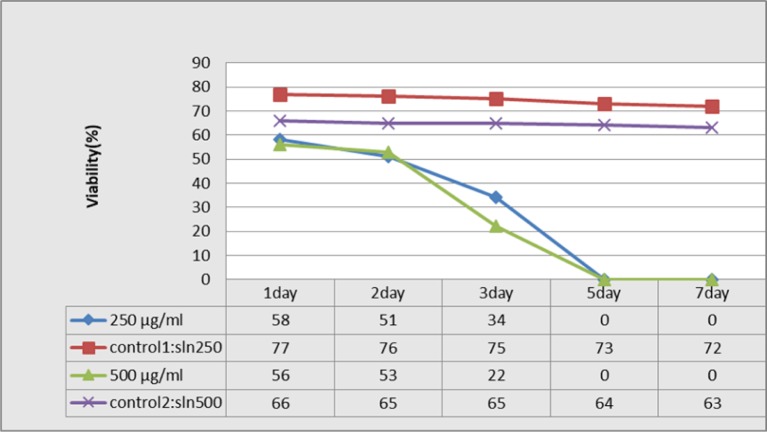
The survival of protoscolex at different time periods and various concentrations of Nano-albendazole

After the application of the desired concentrations of albendazole and nanoalbendazole in the protoscolex culture medium, the protoscolicidal potential of the drugs and controls was examined at different time periods (Figs. 2, 3).

### Evaluation of the pathogenicity of the remaining protoscolices in the culture medium

**Fig. 4:**
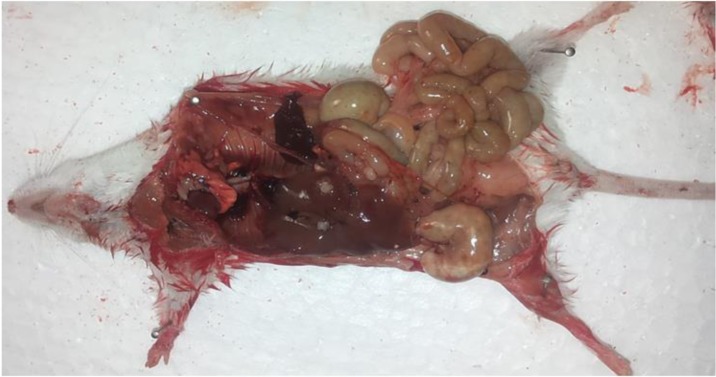
BALB/c mice infected with hydatid cyst growth after intrapritoneally injection of protoscolex

Four groups of eight mice were used for experiment. Protoscolices at the concentration of 250 μg/el on the third day were the specimens considered to be injected into the mice. Only two mice (62.5%) from the albendazole loaded SLNs group did not have any cyst, while in other groups, cysts were performed (Fig. 4).

In the first group that albendazole was used, 11 cysts with a mean cyst size of 1.71 ± 0.54 mm and a mean weight of 10.8 ± 3.1 mg were observed, while in the control group that DMSO + ethanol was used, 17 cysts with the mean size of 2.64 ± 0.79 mm and mean weight of 28.8 ± 9. 27 mg were found.

Of the eight mice in albendezole loaded SLNs group, five mice developed cysts with a mean size of 0.97±0.26 mm and a mean weight of 6.7±1.39 mg. In the control group, SLN was used and after observing 11 cysts, the mean cyst size and weight were calculated to be 2.2 ± 0.65 mm and 18.2 ± 6.03 mg, respectively.

## Discussion

Chemotherapy of hydatid cyst treatment, especially in high-risk cases for surgery, is still a research priority in cystic echinococcosis patients. In addition, the risk of recurrence of post-surgical disease is one of the problems of treatment in patients’ cystic echinococcosis. Therefore, research on protoscolicidal agents which while having no adverse effects on the organs of human body is of paramount importance (27, 28). Several studies have been conducted on the use of albendazole along with surgery and have shown satisfactory results in the treatment of patients (29), but an appropriate formulation to increase the efficacy of the drug has not been found yet. Nano-particles are among the appropriate drug carriers for loading and transferring drugs (30).

In this study, a microemulsion method was used to prepare a SLN carrier. The selected concentrations considered for the current study were 250 and 500 μg/ml for the protoscolicidal property of drugs. The rate of protoscolicidal effect was greater at higher albendazole concentrations, but ultimately the highest rate of protoscolicidal effect was observed on the seventh day of protoscolex exposure in the culture medium. This finding demonstrates the proper effectiveness of protoscolicidal albendazole in vitro conditions.

An albendazole concentration of 50 μg/ml after 30 d could destroy protoscolices (31). In another study on the effect of albendazole on protoscolices, the highest concentration was 200 μg/ml, the highest rate of protoscolicidal effect on the seventh day, and all protoscolices were dead (32). Therefore, albendazole has the highest effect at this concentration, and no significant changes in its protoscolicidal effect will occur at higher concentrations.

However, as with the effect of albendezole in SLN system, the highest rate of protoscolicidal effect occurred on day 5, indicating a greater and faster effect of these nanoparticles than conventional albendazole in the course of their protoscolicidal effect. A study on the protoscolicidal effect of silver nanoparticles under in vitro conditions was conducted and evaluated this drug at different concentrations and time periods. The highest rates of protoscolicidal effect were observed at 0.1 and 0.15 mg/ml after 120 min of exposer, which were 83% and 90%, respectively. Different concentrations of this drug had a high protoscolicidal effect (33).

In order to investigate the pathogenicity of the remaining live protoscolices in a culture medium with a concentration of 250 μg/ml on the third day, infusion was made into the mice. Three months later, after autopsy of the mice, the albendazole group showed contamination in all of the mice, but the weight and number of the cysts decreased in comparison with the control group, indicating the effect of this drug on protoscolices.

Pollat *et al*. from Turkey compared the effect of betadine and albendazole on protoscolices under in vitro and in *vivo* conditions. There were no cysts in the mice injected with protoscolices after exposing protoscolices to 100 μg/ml albendazole (34). The differences results of current study could be attributed to duration of drug efficacy on protoscolices. The lowest cyst size and weight were observed in nono-albendazole experimental group that indicates the effect of this nanoparticle on the prevention process. Moreover, in the nanoalbendazole group, three mice had no cyst development, which indicates higher effectiveness of nano form.

Our result is in agreement with Rafiei et al. research, due to the effectiveness of albendazole on prevention of cyst development. Cysts were observed in only seven mice treated with albendazole, while all the mice in the control group were infected. There was also a major difference regarding the weight and number of cysts (35). The results obtained in these studies were consistent with the results of the present study, the effect of the nano-formulation of albendazole is higher than that of the free form of the drug. Soltani et al. investigated the permeability level of albendazole, albendazole sulfoxide and their loaded form on SLNs to hydatid cyst membranes. Compared with the other two forms, the loaded SLNs drug had more appropriate physico-chemical properties and targeted release, increased permeability and higher efficacy for the treatment of this disease (36).

Another study compared the effects of the combination of albendazole and praziquantel and their loaded form on SLNs on hydatid cyst and found that the loaded form these two drugs as opposed to the free form causes a greater improvement in the disease (25).

Comparison of the results of cyst growth in four groups of mice shows that although the number of cysts grown in the body of the albendazole treated group was equal to that of the SLN control group, the size and weight of these cysts showed a higher protoscolicidal effect of albendazole on protoscolices.

## Conclusion

Albendazole alone can be effective in preventing the development of hydatid cyst if used in vitro with SLNs in sufficient duration and dose to affect protoscolices, it can be used as a suitable drug which can prevent this disease more effectively.

The efficacy of nano-albendazole under in vivo conditions in the present study may be indicative of the future use of this drug. Both time and concentration are important determinants of the effectiveness of these drugs such that by increasing these two factors, the effectiveness of these drugs will be higher, and more satisfactory results will be obtained.
